# Dysfunction of Decidual Macrophages Is a Potential Risk Factor in the Occurrence of Preeclampsia

**DOI:** 10.3389/fimmu.2021.655655

**Published:** 2021-05-12

**Authors:** Miaomiao Rong, Xingyu Yan, Hongya Zhang, Chan Zhou, Cong Zhang

**Affiliations:** ^1^ Shandong Provincial Key Laboratory of Animal Resistance Biology, College of Life Sciences, Shandong Normal University, Ji’nan, China; ^2^ School of Medicine, Xiamen University, Xiamen, China; ^3^ Center for Reproductive Medicine, Ren Ji Hospital, School of Medicine, Shanghai Jiao Tong University, Shanghai, China; ^4^ Shanghai Key Laboratory for Assisted Reproduction and Reproductive Genetics, Shanghai, China

**Keywords:** preeclampsia, decidual macrophages, immune, immunoregulation, immune cell

## Abstract

Preeclampsia is a multi-factorial and multi-genetic disorder that affects more than eight million mother and baby pairs each year. Currently, most of the attention to the pathogenesis of preeclampsia has been focused on placenta, but recent progresses suggest that excellent decidualization lays foundation for placentation and growth. Moreover, preeclampsia is associated with an imbalance in immunoregulatory mechanisms, however, how the immune regulatory system in the decidua affects preeclampsia is still unclear. In our study, after intersecting the genes of differentially expressed between preeclampsia and the control gotten by conventional expression profile analysis and the genes contained in the ligand receptor network, we found eight differentially expressed genes in a ligand-receptor relationship, and the eight genes have a characteristic: most of them participate in the interaction between decidual macrophages and other decidual immune cells. The results of single-cell sequencing of decidual cells further demonstrated that decidual macrophages affect the functions of other immune cells through export. As a result, abnormal gene expression affects the export function of decidual macrophages, which in turn affects the interaction of decidual macrophages with other immune cells, thereby destroying the original immune regulation mechanism, and ultimately leading to the occurrence of preeclampsia.

## Introduction

Preeclampsia (PE) is a pregnancy-related syndrome with high blood pressure (≥ 140/90 mmHg) and/or proteinuria (≥ 0.3 g/L) (1). It is a human-specific disease that can severely cause uteroplacental dysfunction, intrauterine growth restriction, and premature birth, and is one of the major causes of maternal and fetal morbidity and mortality (2). So far, the only and most reliable treatment for PE is delivery, but the pathogenesis of PE is still an enigma. About the pathogenesis, two-stage theory is now widely accepted: In stage I, during embryogenesis, trophoblast invasion of the uterine myometrium is inadequate and the uterine spiral artery remodeling is impaired, which leads toplacental ischemia and hypoxiametabolism disorder; In stage II, the presence of a large number of undesirable factors in the maternal blood leads to a systemic inflammatory response and vascular endothelial dysfunction in the maternal body, which eventually results in PE (3–5).

Recent study in the pathogenesis of PE demonstrated that decidualization is closely related to the occurrence of PE. Decidualization is a transformation that endometrial stromal cells must undergo in order to adapt to pregnancy, by affecting trophoblast invasion, embryo implantation (6). It firstly begins near spiral artery and then gradually spreads to all endometrium (7). Decidualization is an extremely complex process in which many cytokines and hormones cooperate to promote endometrial stromal cells differentiating into decidual cells (8). After decidualization, endometrium transforms into decidua and secretes insulin-like growth factor binding protein 1 (IGFBP1) and prolactin (PRL), both of which are thus recognized as the marker molecules of decidualization, along with the changes such as the thickening of the endometrium and the enlargement and rounding of the endometrial stromal cells (9, 10). Furthermore, the decidua contains a large number of pregnancy-related immunomodulatory cells that work together to form immune tolerance to pregnancy, which makes the decidua becomes an important site where the maternal immune system develops tolerance to fetal antigens. Aberrant frequency and function of decidual immune cells have been reported in a variety of obstetric complications, including PE, recurrent pregnancy loss, and preterm delivery (11, 12).

From an immunological point of view, the fetus is a homograft to the mother. Despite the antigenic differences between the mother and fetus, the maternal immune system does not reject it and protects it to develop normally, indicating that the immune system plays an important role in the normal course of pregnancy (13, 14). Decidual immune cells are important “enforcers” of maternal-fetal immune regulation, including decidual natural killer cells (dNK), decidual macrophages (dM), T cells, and to a lesser extent, dendritic cells (DC) and B cells. These immune cells converge locally in the uterus, forming a unique mother-fetus immune microenvironment (15–18).

As a subpopulation of mononuclear phagocytes of innate immune system, dM is highly plastic and heterogeneous. It can be classified into M1/M2 cells based on induction factors, phenotype and function (19). M1 cells have high expression of CD80, CD86, and MHC II, produce pro-inflammatory factors, and have a strong antigen-presenting ability, thus promoting Th1-type immune responses. M2 cells highly express CD206, CD163, CD209, etc., which produce anti-inflammatory factors and have strong immunosuppressive ability so as to promote Th2-type immune response to exert immunomodulatory function (20). Abnormalities in the phenotype and function of dM would cause an imbalance in the microenvironment at the mother-fetus interface and disruption of immune tolerance, which in turn would lead to dysfunctional trophoblast invasion and defective spiral artery reconstitution, ultimately result in PE (21). But the detailed mechanism by which dM causes PE remains unclear.

In this study, we used the newly obtained single-cell sequencing data to investigate the pathogenesis of PE, and found that the export role of dM was crucial, since most of the differentially expressed genes in the decidua parietalis of PE gotten by intersecting the genes found though expression profile analysis and the genes contained in the ligand receptor network are in the export function of dM, so that the aberrant function of dM is an important cause in the development of PE.

## Materials and Methods

### Data Collection

We downloaded the raw data of single-cell sequencing and the gene expression matrix from Gene Expression Omnibus (GEO, https://www.ncbi.nlm.nih.gov/geo/). The original source of single cell data is GSE130560, which comes from the decidua of three healthy term deliveries. The expression profile of the decidua parietalis of severe PE (early-onset preeclampsia) and control group is derived from GSE94643 in the GEO database. Each group covers four sets of data. The control group had a spontaneous preterm birth with no signs of infection, while the severe PE patients had caesarean sections.

### Data Processing, Quality Control and Standardization

We firstly used the Seurat package in the R software to process the original data count array (22). Then we used the “NormalizeData” function to normalize the gene expression data. Afterwards, the UMI and mitochondrial gene expression values detected by the “ScaleData” function were used to correct the inter-batch difference by regression analysis, and the corrected expression matrix was then used for cell clustering and dimensionality reduction.

### Screening of Differentially Expressed Genes

We downloaded the standardized data-series matrix from GEO, and calculated the differentially expressed genes to be screened by the R language using the T-test (Student’s t-test) statistical method. The screening conditions: p-value <0.05; fold change>1.5 or <0.667.

### Functional Enrichment Analysis of Differentially Expressed Genes

Gene Ontology Database is referred to as GO database. It describes genes and protein functions in detail in a tree-like hierarchical manner to clarify the hierarchical relationship between gene functions. GO Analysis is a method based on the GO database to accurately targeted screen out the gene functions that represent the target gene group significantly. We used GO Analysis method to annotate the gene function of the differential genes, and got all the functions that the gene participates in. Subsequently, we used Fisher’s exact test and multiple comparison test to calculate the significance level (p-value) and false positive rate (FDR) of each function, so as to screen out the notable function embodied by the differential genes. The criteria for significance screening: p-value<0.05.

### Signal Pathway Analysis of Differentially Expressed Genes

Kyoto Encyclopedia of Genes and Genomes (KEGG) is a database that systematically analyzes the relationship between genes, gene functions and genome information, which helps to study genes and expression information as a whole network for research. Pathway analysis was based on the KEGG database and was performed by using Fisher’s exact test and chi-square test for differential genes to analyze the significance of the pathway in which the target gene participates, and screening was done according to p-value<0.05 to obtain significant pathways.

### Ligand-Receptor Pair Database

The ligand-receptor pairs analyzed in this study came from public data sets provided by previous studies (23). The ligand-receptor analysis was performed using the cellphonedb function provided by the cellphonedb database.

### Cell Clustering, Dimensionality Reduction and Visualization

We used the “FindVariableGenes” function in the Seurat software package to extract the eigenvalues of highly variable genes (HVGs) for cell clustering and dimensionality reduction. Then we used the “RunPCA” function to perform principal component analysis on HVGs.

In order to remove the signal-to-noise ratio, we utilized the “ElbowPlot” function to select important principal components. Then, we used the “FindClusters” function to cluster the cells in the PCA space. We set the parameter resolution to 0.8 and only identified main cell types, such as T cells, B cells, or macrophages. We used the “RunUMAP” function to project the clustered cells into a two-dimensional space, and employed “DimPlot” function to realize the visualization of the clustering results.

### Identification of Cell Types

In order to label cell clusters, we first executed the “FindMarker” function through the Seurat software package to identify the differentially expressed genes on each cluster. Then we used the transcriptome data set of the cell types to infer the cell type of each single cell through Single R, and annotate the cell population.

### Analysis of the Interaction Between Cells

We used the cellphonedb function provided by the cellphonedb database (www.CellPhoneDB.org) for cell-cell interaction analysis (24). The threshold for screening was p value<0.01.

## Results

### Differential Gene Expression in the Decidua Parietalis of PE

**Figure 1:**
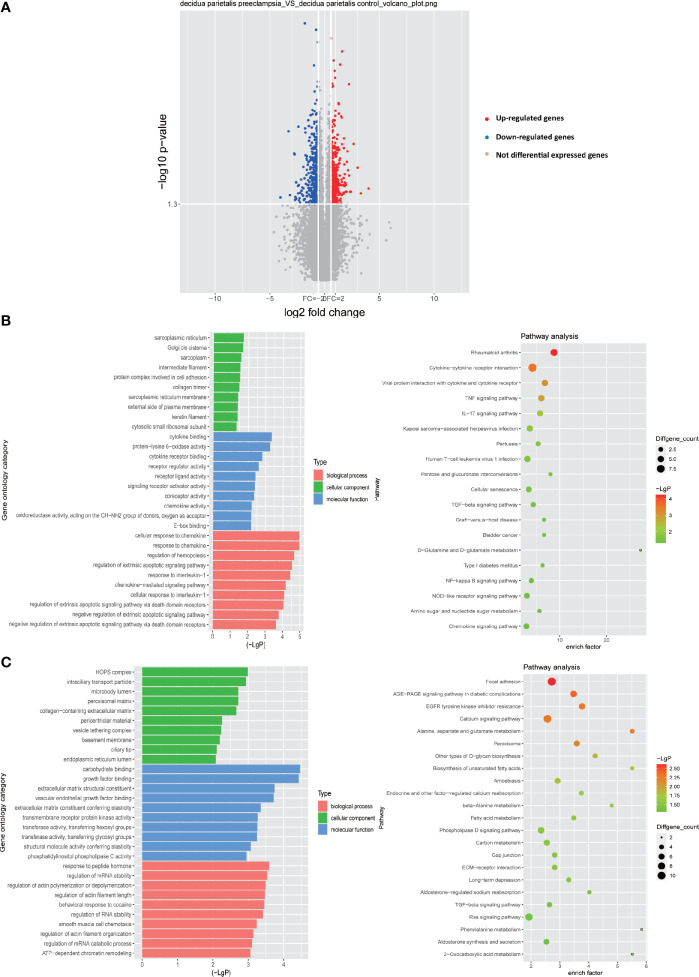
Differential gene expression in the decidua parietalis between PE and control group. **(A)** Volcano figure showing the significantly DEGs in the decidua parietalis of the PE in comparison to controls. A red dot represents up-regulated DEGs with log2 ratio ≥1.5 and p-value <0.05; a green dot represents down-regulated DEGs with log2 ratio ≤ -1.5 and p-value <0.05. **(B)** GO analysis and KEGG pathway enrichment for the down-regulated DEGs. Left panel: GO functional classification of the down-regulated DEGs. Green, blue, and red represent the three classes of the GO term. The top ten enriched GO terms were shown in each class. Right panel: Scatter plot for the KEGG enrichment of the down-regulated DEGs. The enrich factor is the ratio of the down-regulated DEGs in a pathway to all the genes in this pathway. The top 19 pathways were shown with p-value< 0.05 in the figure. **(C)** GO analysis and KEGG pathway enrichment for the up-regulated DEGs. Left panel: GO functional classification of the up-regulated DEGs. Green, blue, and red represent the three classes of the GO term. The top ten enriched GO terms were shown in each class. Right panel: Scatter plot for the KEGG enrichment of the up-regulated DEGs. The top 23 pathways were shown with p-value< 0.05.

So far, more and more studies have shown that abnormal decidualization is an important factor leading to PE (6, 25–30). However, the abnormal interaction between decidual cells based on the ligand-receptor relationship in PE is still unclear. Therefore, by conventional expression profile analysis, we tested the differentially expressed genes (DEGs) in the decidua parietalis of the four groups of PE (GSM2480233-NS, GSM2480234-NS, GSM2480235-NS, GSM2480236-NS) and the normal control group (GSM2480225-NS, GSM2480226-NS, GSM2480227-NS, GSM2480228-NS). The results showed that compared with the control group, the gene expression in the decidua parietalis of PE was significantly different. In detail, 548 genes in the decidua parietalis of PE were significantly up-regulated and 533 genes were significantly down-regulated (**Figure 1A**). In order to better study the genes significantly differentially expressed in PE, we further analyzed the functions of up-regulated and down-regulated DEGs and the signal pathways they are involved through GO and KEGG analysis.

GO analysis of the down-regulated DEGs showed that in terms of biological processes, the most enriched GO item is cellular response to chemokine. In addition, in terms of cell components, the most enriched GO item is sarcoplasmic reticulum. More importantly, protein complex involved in cell adhesion which enriches any protein complex that is capable of carrying out some part of the process of cell adhesion to the cell matrix or to another cell is also abundantly enriched. Moreover, the most abundant GO item in molecular functions is cytokine binding. Receptor ligand activity and chemokine activity are also significantly enriched (**Figure 1B**). Through the KEGG signal pathway analysis of the down-regulated DEGs, 19 signal pathways were found to be significantly enriched. Among them, the most abundant signaling pathway is cytokine-cytokine receptor interaction. Besides, the TNF signaling pathway, IL-17 signaling pathway, TGF-beta signaling pathway, NF-kappa B signaling pathway and chemokine signaling pathway are also significantly enriched (**Figure 1B**).

Through GO analysis of the up-regulated DEGs, it is obvious that in terms of molecular function, carbohydrate binding is most significantly enriched, while extracellular matrix structural constituent, growth factor binding and transmembrane receptor protein kinase activity are also significantly enriched in molecular function. In terms of cellular components, HOPS complex is the most enriched. Basement membrane is also significantly enriched in cell components, which is consistent with previous our research results. In addition, in terms of biological processes, the regulation of mRNA stability and the regulation of actins polymerization or depolymerization are significantly enriched (**Figure 1C**). We also analyzed the up-regulated DEGs by KEGG signaling pathway and found that among 23 significantly enriched signaling pathways, focal adhesion is the most significant enrichment, and calcium signaling pathway, TGF-beta signaling pathway, phospholipase D signaling pathway and Ras signaling pathway are also significantly enriched.

Overall, these results indicate that the down-regulated DEGs are mainly concentrated in chemokines and immune-related pathways. The up-regulated DEGs may play an important function in the extracellular matrix. As we all know, there are abundant regulatory factors with immunomodulatory activity in the extracellular matrix, such as growth factors (31) and chemokines (32). These factors are closely related to the cell-cell interaction of immune cells. Therefore, the results of the study indicate that these DEGs play a vital role in the immune regulation process in the decidua.

### Abnormal Function of Decidual Macrophages in PE

**Figure 2:**
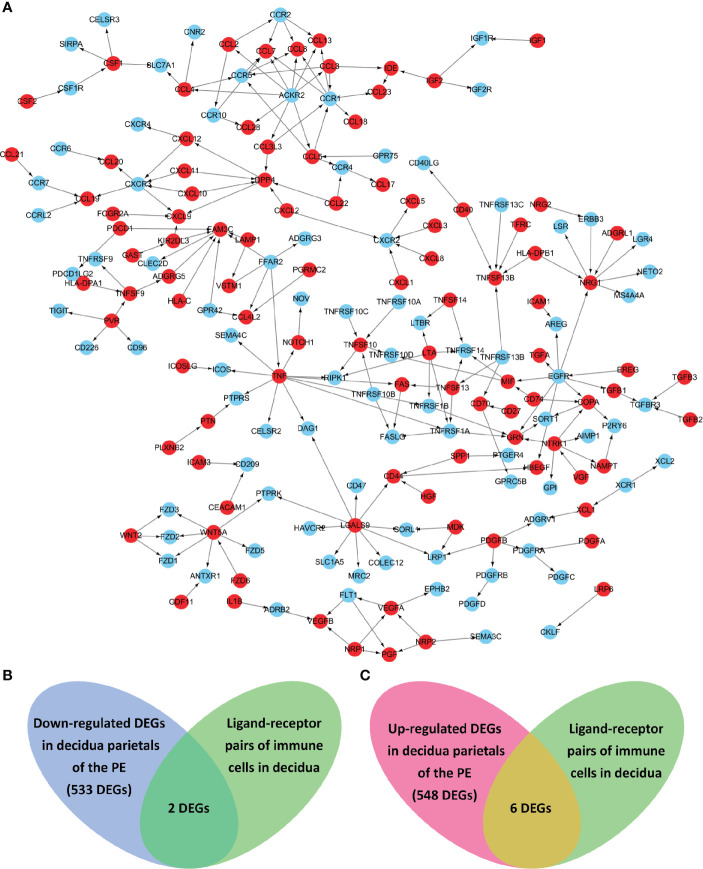
The ligand-receptor relationships of the differentially expressed genes (DEGs) in the decidua parietalis of PE. **(A)** The ligand-receptor signaling network of immune cells in deciduas. Red dots represent ligands and blue dots represent receptors. The arrows points from ligands to receptors. **(B)** Venn diagrams of the significantly down-regulated DEGs in the decidua parietalis of the PE and in the ligand-receptor pairs of immune cells in deciduas. **(C)** Venn diagrams of the significantly up-regulated DEGs in the decidua parietalis of the PE and in the ligand-receptor pairs of immune cells in decidua.

Known from the above results, in the decidua parietalis of PE patients, DEGs affect the cell-cell interaction of immune cells. Therefore, we further studied the cell-to-cell interaction which was specifically affected by the abnormal expression of the differential genes. Cell populations form a potential cell-cell interaction network among specific protein complexes, which are based on the relationship between receptor and ligand genes (**Figure 2A**). Therefore, we compared the genes contained in down-regulated DEGs and up-regulated DEGs and in the ligand-receptor pairs that interact with immune cells, and found that 2 down-regulated DEGs (ICAM1 and CXCL3) and 6 up-regulated DEGs (IGF1, CXCL12, NRP1, LRP6, PDGFD and PDGFRB) are expressed in a ligand-receptor relationship (**Figures 2B, C**).

**Figure 3:**
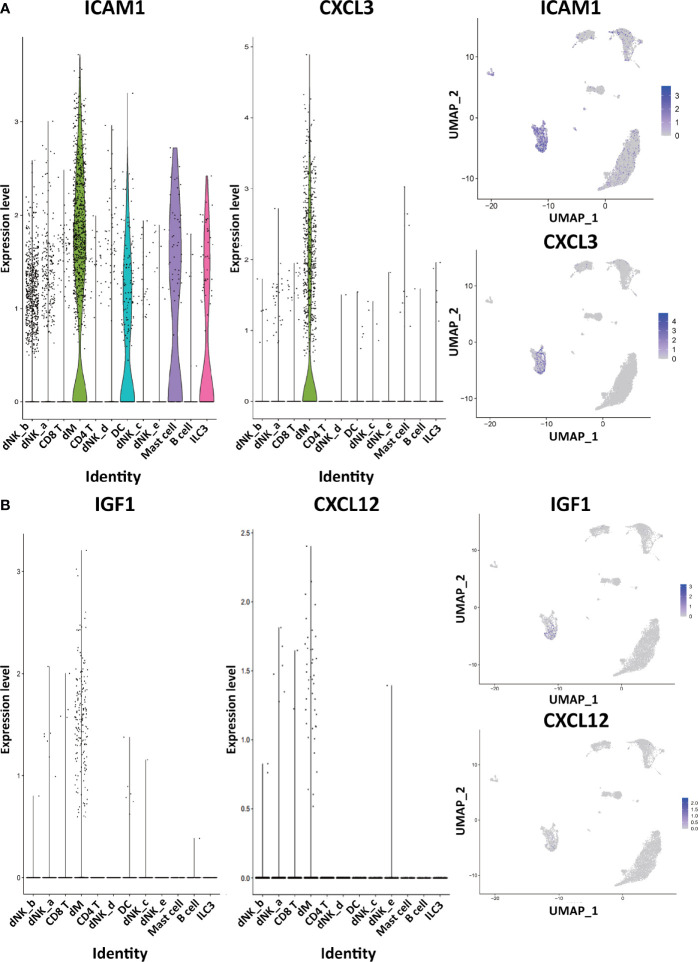
The expression of DEGs in the immune cells of normal decidua. **(A)** The violin plots and the UMAP visualization maps showing the expression of two down-regulated DEGs (ICAM1 and CXCL3) in the immune cells of normal decidua. **(B)** The violin plots and the UMAP visualization maps showing the expression of two up-regulated DEGs (IGF1 and CXCL12) in the immune cells of normal decidua. dM, decidual macrophages; dNK, decidual natural killer cells; ILC, innate lymphoid cells; DC, dendritic cells.

**Figure 4:**
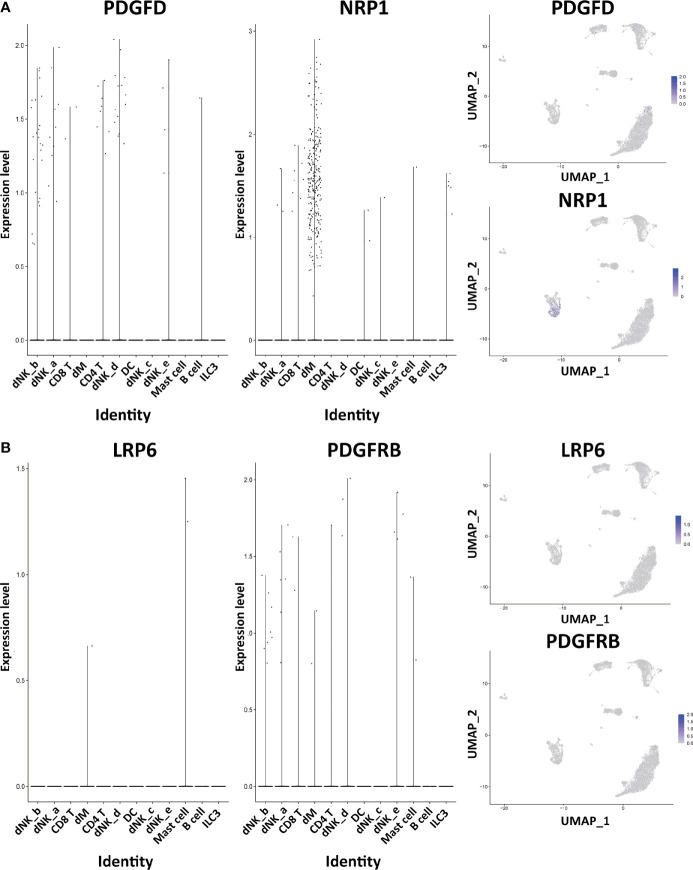
The expression of DEGs in the immune cells of normal decidua. **(A)** The violin plots and the UMAP visualization maps showing the expression of two up-regulated DEGs (PDGFD and NRP1) in the immune cells of normal decidua. **(B)** The violin plots and the UMAP visualization maps showing the expression of two up-regulated DEGs (LRP6 and PDGFRB) in the immune cells of normal decidua. dM, decidual macrophages; dNK, decidual natural killer cells; ILC, innate lymphoid cells; DC, dendritic cells.

**Figure 5:**
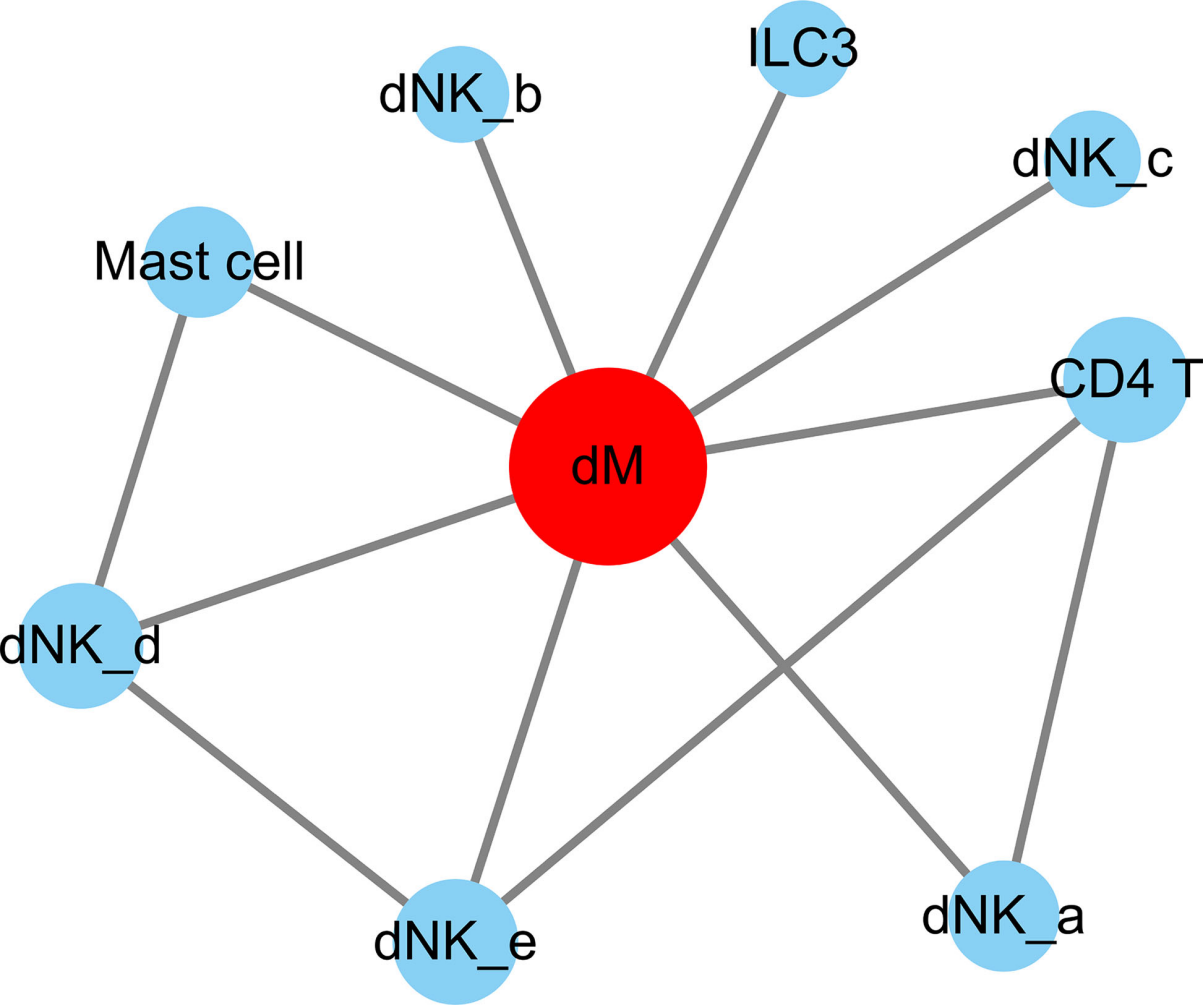
The cell-to-cell interactions of DEGs involved. The nodes are cell types and the segments are cell-to-cell interactions. The red node indicates cell types that are highly interacted with other cells. The size of the different cell types is proportional to the amount of total number of interactions with the red node. dM, decidual macrophages; dNK, decidual natural killer cells; ILC, innate lymphoid cells.

Through the analysis of single-cell sequencing data, we found that ICAM1 and CXCL3 are both expressed in normal dM, but IGF1, CXCL12, NRP1, LRP6, PDGFD and PDGFRB are not expressed in normal dM, nor are they expressed in other immune cells (**Figures 3**, **4**). Then, based on the ligand-receptor relationship that these DEGs participate in, we obtained the cell-to-cell interaction (**Table 1**) and found one characteristics: most of them exist in the interrelationship between dM and other decidual immune cells (**Figure 5**), which indicates that abnormal gene expression affects the interaction of dM with other decidual immune cells.

**Table 1:** The cell-to-cell interactions of differentially expressed genes involved.

inter	interacting_pair	inter	ligand	receptor
CXCL12_CXCR3	dM|dNK_d	0.388	CXCL12	CXCR3
CXCL12_CXCR3	dNK_e|dNK_d	0.37	CXCL12	CXCR3
CXCL12_CXCR4	dM|dNK_d	1.365	CXCL12	CXCR4
CXCL12_CXCR4	dM|CD4 T	1.298	CXCL12	CXCR4
CXCL3_CXCR2	dM|ILC3	0.49	CXCL3	CXCR2
CXCL3_CXCR2	dM|dNK_e	0.49	CXCL3	CXCR2
CXCL3_CXCR2	dM|dNK_e	0.49	CXCL3	CXCR2
CXCL3_CXCR2	dM|Mast cell	0.49	CXCL3	CXCR2
DPP4_CXCL12	CD4 T|dNK_e	0.103	DPP4	CXCL12
DPP4_CXCL12	CD4 T|dNK_a	0.097	DPP4	CXCL12
ICAM1_AREG	dM|dNK_d	1.533	ICAM1	AREG
ICAM1_AREG	dM|dNK_b	1.475	ICAM1	AREG
IGF1_IGF1R	dM|dNK_e	0.135	IGF1	IGF1R
IGF1_IGF1R	dM|dNK_d	0.134	IGF1	IGF1R
LRP6_CKLF	dM|dNK_a	0.975	LRP6	CKLF
LRP6_CKLF	dM|dNK_c	0.962	LRP6	CKLF
NRP1_PGF	dM|ILC3	0.194	NRP1	PGF
NRP1_PGF	dM|dNK_b	0.187	NRP1	PGF
NRP1_VEGFA	dM|Mast cell	0.47	NRP1	VEGFA
NRP1_VEGFA	Mast cell|dM	0.45	NRP1	VEGFA
NRP1_VEGFB	dM|dNK_a	0.209	NRP1	VEGFB
NRP1_VEGFB	dM|ILC3	0.207	NRP1	VEGFB
PDGFRB_PDGFD	dNK_e|dNK_d	0.077	PDGFRB	PDGFD
PDGFRB_PDGFD	Mast cell|dNK_d	0.056	PDGFRB	PDGFD

dM, decidual macrophages; dNK, decidual natural killer cells; ILC, innate lymphoid cells; DC, dendritic cells.

### The Cell-to-Cell Interaction of Decidual Macrophages

**Figure 6:**
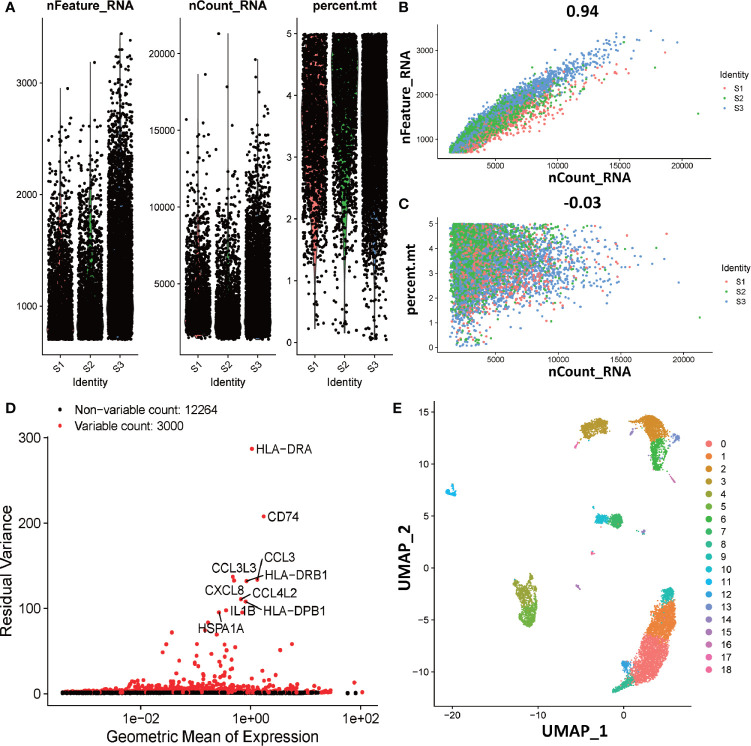
Overview of preparations for cell type identification in deciduas. **(A)** Gene expression in each of the 9066 cells in three normal samples (S1, S2, and S3). The left graph shows the number of genes contained in each cell. The middle figure indicates the confidence level of the data. The right graph shows the percentage of mitochondria. The black dots represent cells. **(B)** The relevance of the number of UMI and the number of genes. The horizontal coordinate is the number of UMI of the cell and the vertical coordinate is the number of genes in the corresponding cell. The red, green and blue dots respectively represent samples S1, S2 and S3. **(C)** The relevance of the number of UMI and the percentage of mitochondria. The horizontal coordinate is the number of UMI of the cell and the vertical coordinate is the percentage of mitochondria. The red, green and blue dots respectively represent samples S1, S2 and S3. **(D)** The analytical map of genetic variability. Red dots represent variable counts and black dots represent non-variable counts. **(E)** The UMAP visualization maps of 19 cell clusters in the deciduas by single cell RNA sequencing analysis.

To explore the function of dM, we further investigated the interaction of dM with other decidual immune cells using single-cell sequencing. The single cell data in decidua comes from the GEO database, which contains three normal samples, corresponding to 9066 cells. In order to standardize the data, we performed data quality control and deleted some cells with expression genes less than 200 and greater than 6000 (UMI count greater than 0 and greater than 6000) and the percentage of mitochondrial genes greater than 5% (**Figure 6A**). As the amount of sequencing data increases, the number of genes detected in a single cell increases and the number of UMIs also increases, indicating that there is a positive correlation between genes and UMI (**Figure 6B**). However, there is no correlation between the number of genes and the percentage of mitochondria, which means that the number of genes is not affected by the percentage of mitochondria (**Figure 6C**). After the correction of the expression matrix, the number of non-variable genes was 12264, while the number of mutant genes was 3000, therefore, the number of mutant genes was far less than the number of non-variable genes (**Figure 6D**). We selected 2000 HVGs from the corrected expression matrix and extracted their feature values for cell clustering and dimensionality reduction. After the principal component analysis of 2000 HVGs, 19 principal components were finally determined through permutation test. As we all know, each type of cell contains a different number and type of genes. Therefore, according to this characteristic, the principal components of the cells were analyzed and projected into the two-dimensional space to achieve the purpose of cell dimensionality reduction. The results showed that cells with the same characteristics will eventually be gathered together, and on the contrary, cells without the same characteristics will be far apart (**Figure 6E**).

**Figure 7:**
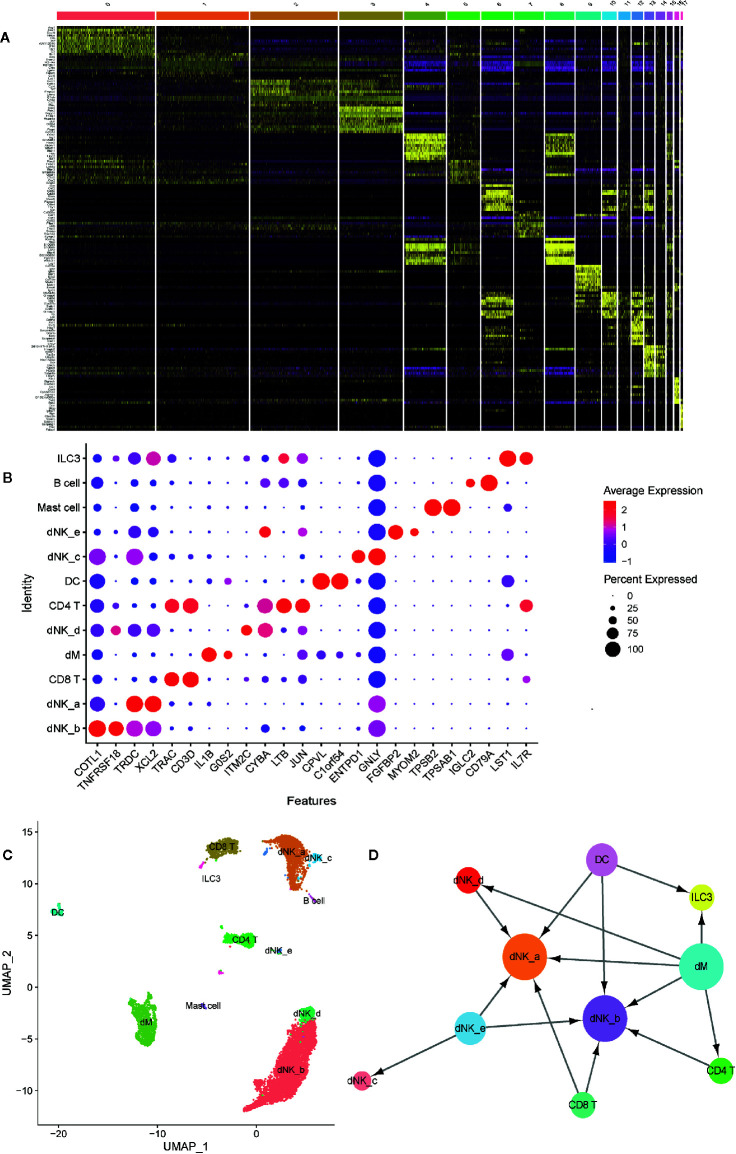
The identification and interactions of decidual cell clusters by single-cell sequencing. **(A)** The heatmap showing the expression of differential genesin separate cell clusters of normal deciduas. **(B)** The dot plot reveals the expression of some known markers in each immune cell clusters of normal deciduas. The size of the ball represents percent expressed. The color of the ball represents average expression. **(C)** The UMAP visualization maps of various immune cell clusters in the normal deciduas by single cell RNA sequencing analysis. The various colors represent different types of cells. **(D)** The cell-to-cell interaction analysis was conducted by CellPhoneDB. Cell types are presented by nodes and interactions are indicated by arrows. The size of the nodes represents the number of cells contained in each cell type in the deciduas (p-value < 0.01). →, export function; ←, import function. dM, decidual macrophages; dNK, decidual natural killer cells; ILC, innate lymphoid cells; DC, dendritic cells.

In order to further investigate the type of cells, we identified the DEGs in each cell population (**Figure 7A**), and selected known cell marker genes from them. Under the influence of biological background, by consulting the literature, we can explore the related genes of each cell type and their expression in different cell types. We found that several cell markers are specifically expressed in only one cell, such as FGFBP2 and MYOM2 are only expressed in dNK-e cells; TPSB2 and TPSAB1 are only expressed in Mast cells; IGLC2 and CD79A are only expressed in B cells. In addition, other cell markers are expressed in a variety of cells, but their expression levels are higher in one cell (**Figure 7B**).

Therefore, we used known cell marker genes expressed in different cell types to define each cell cluster. We combined cell populations expressing the same cell markers and observed that dNK cells are the most abundant in decidua, and dM cells are second only to dNK cells and CD8 T cells. In addition, there are fewer B cells, mast cells, and ILC3 cells (**Figure 7C**). In order to systematically study the interaction between various immune cells in the decidua, we used CellPhoneDB to analyze the interaction between them. The results showed that dM plays a role through the export function and interacts most closely with other immune cells. Abnormal dM can affect the functions of other cells, such as ILC3 cells, dNK-a cells, dNK-b cells, dNK-d cells, and CD4T cells, while the functions of dNK-a cells and dNK-b cells are affected by a variety of cells (**Figure 7D**). In summary, the results indicate that the interaction between the dM and other immune cells plays an important role in the establishment and maintenance of the entire pregnancy process. Therefore, the abnormal export of dM affects the role of immune cells interplaying with them, consequently jeopardizing the original immune regulation, and ultimately causing the occurrence of PE.

## Discussion

In this study, we used conventional expression profiling to compare the DEGs contained in decidua parietalis of the PE and normal human. We further explored their involvement in cell-to-cell interactions using CellPhoneDB analysis. We found that most of differential genes exist in the interrelationship between decidual macrophages and other decidual immune cells. We further used single-cell sequencing and discovered that dM affect the functions of other immune cells in the decidua through exporting function. So, the abnormal exportation of dM due to abnormal expression of differential genes affects the role of immune cells interplaying with them, consequently impeding the original immune regulation, and ultimately causing the occurrence of PE.

Most previous studies on the pathogenesis of PE have been focused on placenta, including placental ischemia and placental dysfunction. However, decidualization is the foundation for placentation and growth. Abnormal decidualization of the endometrium can lead to embryo implantation failure or abnormal implantation, which in turn leads to the occurrence of PE (8, 9). In this process, there are a series of immune regulatory mechanisms. Thereinto, both tumor necrosis factor (TNF) and nuclear factor-κB (NF-κB) are situated at the central link in the network of immune regulation (33, 34). The TNF is a central polypeptide mediator of the cellular immune response, it participates in TNF signaling pathway, and can recognize and participate in the activation of NF-κB (35, 36). In PE patients, the balance of NF-κB signaling pathway is disrupted, leading to abnormal apoptosis of placental trophoblast cells (37, 38). In this research, we found that CXCL3 and ICAM1 participate in the TNF signaling pathway and NF-κB signaling pathway. In addition, these two genes are highly expressed in normal dM, but are significantly down-regulated in PE. Moreover, ICAM1 is also expressed in DCs, mast cells, and ILC3s, but it is not expressed in other decidual immune cells, while CXCL3 is not expressed in other decidual immune cells except for its high expression in dM. At the same time, we also found that the other six genes that are significantly up-regulated in the decidua parietalis of PE are not only not expressed in dM, but also not expressed in other normal decidual immune cells, including IGF1, CXCL12, NRP1, LRP6, PDGFD, and PDGFRB. But they are all expressed in decidual stromal cells, the most numerous cell type in the decidua and an important central player in the recruitment and differentiation of the decidual immune cells (39, 40). The abnormal expression of these DEGs therefore affects the function of the decidual stromal cells and thus the interactions between immune cells in the decidua.

Previous studies have demonstrated that CXCL3 and CXCL12 are chemokines, they can coordinate the migration, positioning and the interaction between immune cells (32, 41). In addition, CXCL3 can inhibit the proliferation of T cells and increase the apoptosis of T cells, while IGF1 has the opposite effect (42, 43). In detail, IGF1 can inhibit T cell apoptosis, and promote T cell autophagy (43). CXCL12 activates PKA, CREB, Bcl2 and BclXl, thereby prolonging the survival time of CD4T lymphocytes. In addition, it promotes the accumulation of NK cells in the uterus by enhancing the adhesion of NK cells to decidual stromal cells (42, 44). ICAM1 is a single-chain transmembrane glycoprotein that can promote the proliferation of naive CD4T cells and also plays a key role in the interaction between antigen presenting cells and T cells (45). In laboring samples, macrophages are recruited as monocytes from the circulation and activated by prostaglandins, while the expression of ICAM1 is central to this process, which allows the attachment and tethering of immune complexes in the periphery (46–48). Our research further demonstrated that the expression of ICAM1 is significantly down-regulated in PE, which may also be an important factor in the occurrence of PE. NRP1 can regulate the transfer of CD4T cells and allow the acceptance of allogeneic grafts, making NRP1 a new target for immunotherapy (49). It is worth noting that the Wnt signaling pathway which LRP6 is involved in regulates the differentiation and development of various immune cells such as macrophages, T cells and B cells, and regulates the immune response process through various mechanisms (50–53). Overall, the above-mentioned DEGs play an important role in the immune regulation. The abnormal expression of these DEGs can cause the disorders of the immune system.

Interestingly, we found that these DEGs have one thing in common: according to the ligand-receptor relationship network, these DEGs are all present in the export function of dM. As the second largest type of leukocyte at the maternal-fetal interface, dM exerts anti-inflammatory and phagocytosis effects in the early pregnancy, and by regulating the local immune microenvironment of the decidua, remodels the decidual blood vessels, participates in embryo implantation and maintains pregnancy and other processes (22, 54). In the decidua of PE patients, the increased expression of TGF-β3 activates miR-494 in decidual mesenchymal stem cells, thereby weakening the regulation of mesenchymal stem cells, thereby turning macrophages into M2 type, and finally leads to the immune imbalance of maternal-fetal interface (55). Imbalanced ratio of M1/M2 macrophages is considered to be one of the causes of pregnancy-related diseases, including PE, fetal growth restriction (FGR) and preterm delivery (18, 56). Our research found that dM affects the function of ILC3 cells, dNK-a cells, dNK-b cells, dNK-d cells, and CD4T cells through export in the decidua. Therefore, the abnormal export of dM affects the function of immune cells that interact with it, thereby destroying the original immune regulation mechanism.

Although dM is not the focus of most studies on the pathogenesis of PE, our research has found that DEGs that appear in the export of dM play a key role in the development of PE. To be precise, the activation of NRP1 can affect angiogenesis, and it activates the intracellular kinase ABL1 independently of the VEGF signaling pathway (57). Whereas abnormal angiogenesis is an important factor leading to PE (58). LRP6 regulates the autophagy mediated by Rab7, and then regulates the invasion and migration of trophoblasts through the Wnt/β-catenin pathway (50, 51). Insufficient invasion of extravillous trophoblast can lead to impaired uterine spiral artery remodeling and subsequent PE (59). IGF1 is one of the genes related to the insulin signaling pathway, and it’s up-regulation may cause polycystic ovary syndrome and endometrial cancer (60). However, in PE, the impact of IGF up-regulation is unclear.

Since no one can obtain a pregnant woman’s decidua during her pregnancy, the samples analyzed were collected at the time of delivery. Although we cannot know the real state of the decidua in the first trimester of pregnancy when the placenta is formed, many studies have found that numerous decidualization-related genes are abnormally expressed in the decidua of PE patients (28, 61–65). Exploring the relationship between impaired decidualization and PE revealed that the maturity of the endometrium in the decidua and the natural killer cells of the uterus before the development of PE did not reach the ideal level (66). Moreover, decidualization defects of the endometrium in women with severe PE were found during delivery, which was characterized by impaired cytotrophoblast invasion, and could last for at least five years after conception (25, 67). Recently, it was discovered that insufficient expression of maternal annexin A2 expression can lead to aberrant decidualization and superficial cytotrophoblast invasion (68), suggesting that poor differentiation of endometrial cell during the menstrual cycle could underlie superficial trophoblast invasion and the poor establishment of the maternal-fetal interface. On the basis of these findings, the transcriptional signature in the endometrium that promotes decidualization deficiency could be detected before or after pregnancy (67). In addition, the control samples of PE study were from laboring deliveries of gestationally matched preterm births, while the PE samples were from caesarean sections. It is likely that the macrophages are activated in the laboring samples and thus the transcriptomic profile of the control group may be skewed to an inflammatory state associated with labor. This might have some impact on the results of this study.

In summary, our research has found that the imbalance of immune regulation caused by abnormal dM function plays an important role in the pathogenesis of PE. These findings provide new insights for studying the pathogenesis of PE, and also give new ideas for the diagnosis and treatment of PE. However, how do CXCL3, ICAM1, IGF1, CXCL12, NRP1 and LRP6 as ligands or receptors affect the export function of dM, which in turn affects the function of other decidual immune cells, thereby destroying the original immune regulation mechanism, and ultimately leading to the occurrence of PE, which needs to be further investigated.

## Data Availability Statement

The datasets presented in this study can be found in online repositories. The names of the repository/repositories and accession number(s) can be found in the article/supplementary material.

## Author Contributions

Study design: CoZ. Data analysis: ChZ, XY, HZ, and MR. Data interpretation: MR, XY, HZ, ChZ, and CoZ. Drafting of the manuscript: MR. Revision of the manuscript: CoZ. All authors contributed significantly to this work and agreed to be accountable for the work. All authors contributed to the article and approved the submitted version.

## Funding

This study was supported by grants from the National Key R&D Program of China (2017YFC1001403, 2019YFA0802600), and NSFC (31871512 and 31671199) to CoZ. Support was also obtained by the Shanghai Commission of Science and Technology (17DZ2271100).

## Conflict of Interest

The authors declare that the research was conducted in the absence of any commercial or financial relationships that could be construed as a potential conflict of interest.
